# Longest Survival of Expectantly Managed Twin Gestation Complicated by Previable Preterm Premature Rupture of Membranes at 13 Weeks’ Gestation

**DOI:** 10.7759/cureus.16464

**Published:** 2021-07-18

**Authors:** Kaci Axelson, Muhammad Osto, Rafey Rehman, Mona Fakih, Theodore Jones

**Affiliations:** 1 Department of Obstetrics and Gynecology, Beaumont Health, Dearborn, USA; 2 Department of Dermatology, Wayne State University School of Medicine, Detroit, USA; 3 Department of Obstetrics and Gynecology, Beaumont Health, Royal Oak, USA

**Keywords:** dichorionic diamniotic twins, pregnancy, rupture of membranes, preterm premature, obstetrics

## Abstract

Previable preterm premature rupture of membranes (PV-PPROM) is defined as rupture of membranes prior to 24 weeks and is a rare phenomenon with an estimated prevalence of 0.5% of all pregnancies. Given that this phenomenon is even more rare in patients with dichorionic diamniotic (DCDA) twin pregnancies, there is no clear consensus in the literature on outcomes and management of DCDA PV-PPROM due to the scarcity of reports. We report a case of a rare successful prolongation of first trimester DCDA PV-PPROM pregnancy with rupture of the amniotic sac of one twin with survival of both twins without major complications. A 20-year-old female gravida 1 para 0 at about 13 weeks and three days presented with vaginal watery discharge mixed with vaginal bleeding. Abdominal ultrasound revealed a live twin dichorionic diamniotic (DCDA) spontaneous intrauterine gestation and a significantly low amniotic fluid volume involving fetus A. At 23 weeks gestational age, she experienced increased leaking of clear fluid, and she was admitted to the hospital for continuous monitoring with daily non-stress tests (NST), and ultrasounds every four weeks, and received antibiotics, betamethasone, and magnesium. Preterm labor occurred at 30w1d, and a primary low transverse cesarean section was performed on the 114th day after PPROM. Though, twin A required prolonged hospitalization both twins recovered and progressed well without complications. To the best of our knowledge, this is the longest case of successful expectant management of both twins with PV-PPROM yet reported.

## Introduction

Preterm premature rupture of membranes (PPROM) is a relatively common complication of pregnancy, affecting approximately 3%-4.5% of deliveries and poses a high risk fetal and maternal morbidity and mortality. Furthermore, PPROM has a higher rate and shorter latency from the time to PPROM to delivery in twins compared to singletons [1,2]. Previable preterm premature rupture of membranes (PV-PPROM) is defined as spontaneous or iatrogenic rupture of membranes (ROM) before 24 weeks and is quite rare with an estimated occurrence in 0.5% of all pregnancies. Previable PPROM in dichorionic diamniotic (DCDA) twin pregnancies are even more rare, and there is no consensus in the literature on outcomes and management due to scarcity of reports. Most complications are due to prematurity or prolonged rupture of membranes (ROM), such as increased pulmonary hypoplasia, chorioamnionitis or placental abruption [3]. Management options have included either termination of pregnancy (TOP), selective termination of fetus with ruptured sac, or expectant management [3]. Though termination of pregnancy is noted to lead to favorable maternal outcomes, it may not be an acceptable option for patients opposed to termination due to religious beliefs or emotional reasons. Previously, a retrospective cohort study reported higher survival rates up to 82% in selective reduction of one twin [3]. In contrast, one study evaluating expectant management in pre-viable pregnancies with ROM estimated a survival rate as low as 18% [4]. However, a recent case series showed an 83% survival rate among expectantly managed DCDA PV-PPROM [5]. Though maternal chorioamnionitis and sepsis are a concerning complication, median latency is variable and may average less than two weeks. We report a rare case of successful prolongation of first trimester DCDA PV-PPROM pregnancy without any complications and discuss what made this possible. To the best of our knowledge, this is the longest case of successful expectant management of both twins with PV-PPROM. 

## Case presentation

**Figure 1:**
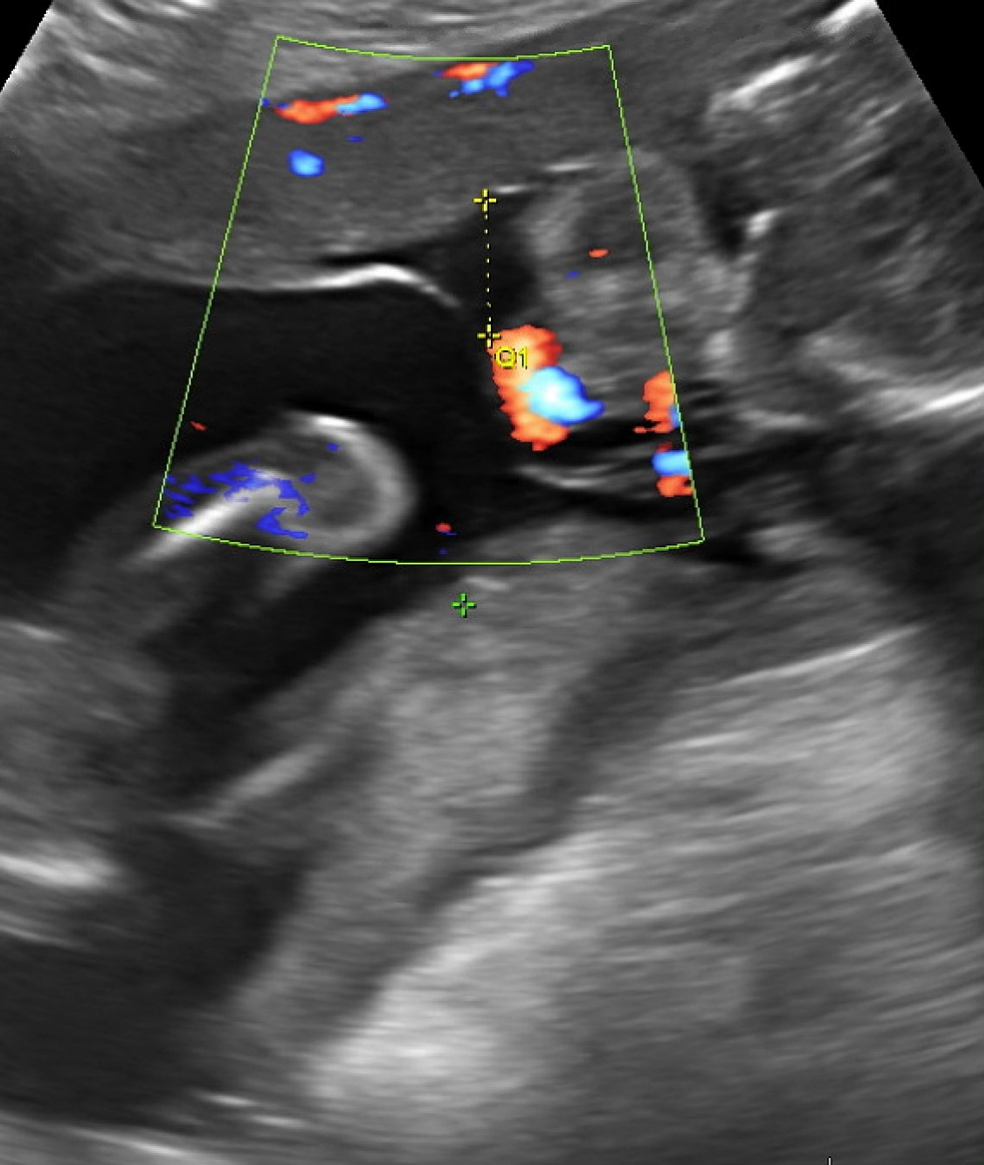
Diamniotic dichorionic twin pregnancy at 19 weeks and 4 days gestation with low amniotic fluid volume involving fetus A as indicated here by a maximum vertical pocket of 1.51 cm.

A 20-year-old pregnant woman (gravida 1, para 0 at approximately 13 weeks and three days) presented to her obstetrician’s office with watery vaginal discharge mixed with vaginal bleeding. She had no pertinent past medical or surgical history. An abdominal ultrasound revealed a live twin DCDA spontaneous intrauterine gestation and a significantly low amniotic fluid volume involving fetus A but an acceptable amount of amniotic fluid in Fetus B, with heart rates of 163 beats per minute (bpm) and 168 bpm, respectively (Figure 1). The patient was advised to be on pelvic rest and told to go to the emergency department (ED) if there was a worsening or recurrence of her vaginal bleeding. The following week, the patient presented to the ED with similar watery, blood-tinged vaginal fluid and abdominal discomfort. Abdominal ultrasound findings were unchanged from the previous study, and her care team diagnosed her with PV-PPROM. The patient was extensively counseled and chose to manage expectantly with weekly outpatient follow-up visits pending signs of infection or maternal compromise. The patient’s pregnancy was monitored via serial abdominal ultrasounds during office visits that demonstrated live twin pregnancies with continued anhydramnios of Twin A. At 23 weeks' gestational age, she experienced increased leaking of clear fluid, and she was admitted to the hospital for continuous monitoring with daily nonstress tests (NST), ultrasounds every four weeks, and a regimen of antibiotics, betamethasone, and magnesium. Latency antibiotics regimen at admission included azithromycin 1 gram once orally, ampicillin 2 gram intravenous every six hours for two days, followed by an additional five days of amoxicillin 500 milligrams three times daily. During this time, the patient experienced no abdominal discomfort, odorous discharge, or other signs of infection. Abdominal ultrasounds revealed similar findings as before with a finding of live twin pregnancies. While serial ultrasonographic examinations showed an initial delay in the growth of Twin A, it approached normal growth by 27 weeks and showed no obvious signs of pulmonary hypoplasia. A series of NST results were reactive.

Moreover, there was evidence of Twin A producing amniotic fluid as it was able to maintain a single maximal vertical pocket (MVP) around 2.0 cm despite vaginal leakage of fluids, which was reassuring. The patient was given labetalol for treatment of sinus tachycardia. On day 113 post-PPROM, at 30 weeks, zero days (30w0d), the patient reported concerns of uterine contractions every four minutes with a pain rated as 5/10 on the analog pain scale. Intravenous betamethasone and magnesium sulfate were given, but no additional tocolytics were given. Preterm labor occurred at 30w1d, and a primary low transverse Cesarean section was performed on the 114th day after PPROM. Twin A was a male baby, weighing 1520 g, with an appearance, pulse, grimace, activity, respiration (APGAR) score of seven and eight. Twin B was also a male baby, weighing 1650 g, and had an APGAR score of five and eight. Pathologic examination of the placenta showed no signs of chorioamnionitis. The postoperative course was complicated by endometritis, for which the patient was treated with antibiotics. The patient was discharged after three days. Twin A was admitted to the neonatal intensive care unit (NICU) after showing signs of severe distress and retractions with poor air exchange indicative of pulmonary hypoplasia and was electively intubated. After five days, he was transitioned to nasal continuous positive airway pressure (NCPAP) for two weeks and then transitioned to a high-flow nasal cannula along with furosemide twice daily for four days, then once daily for two months. He was also given spironolactone, chlorothiazide, and budesonide inhaler daily during his hospital course. He also received two periods of 10-day hydrocortisone taper, one month apart. Twin A was discharged after three months and 15 days, showing marked improvement. Twin A was improving and stable on 0.5 L supplemental oxygen, chlorothiazide, and spironolactone. He also received palivizumab.

Twin B was also admitted to the NICU for prematurity and respiratory distress treated with rescue surfactant and NCPAP, which was discontinued after two days. Then he was transitioned to breast milk and room air with occasional episodes of hypoxia, needing 1 L of nasal cannula oxygen. Twin B was discharged after one month and was doing well without any complications.

## Discussion

A case of PV-PPROM at 13 weeks' gestation and survival of both infants in a DCDA twin pregnancy is described. PV-PPROM is a rare condition, and pregnancies affected have high perinatal and neonatal morbidity and mortality rates. In our case, the outcome was more favorable than we would have anticipated from prior experience and given the current literature. For example, we know that the duration of the latency period appears to be inversely related to the gestational age at which rupture occurred. In a retrospective cohort study of 59,935 deliveries, the mean latency period was 3.3 days in women presenting at 30 to 33 weeks and 14.6 days in those presenting at 23 to 26 weeks [6]. In our case, the latency period was 114 days and appeared to follow or exceed expectations for the inverse relationship described.

Studies have shown that the etiology of PV-PPROM and preterm labor is likely a more general process of intrauterine inflammation (i.e., chorioamnionitis). However, there is also new evidence suggesting that up to half of the cases may also be related to abnormal trophoblastic development in the late first trimester [7]. As our patient never showed any signs of intrauterine infection, the cause for rupture may have been related to the latter. Dysfunctional trophoblastic development is more traditionally associated with preeclampsia. As our patient delivered at 30 weeks, it is unknown if preeclampsia would have eventually emerged if the pregnancy would have progressed longer. It should be recognized that our patient had a history of chronic hypertension, albeit well-controlled.

In a retrospective study of multifetal pregnancies with PV-PPROM, overall neonatal survival at discharge was 43%, and only 17% survived without significant neonatal morbidity [8]. The risk of fetal death appears to be inversely related to the gestational age at PROM. All fetal deaths in one series of studies occurred in pregnancies with MVP less than 2 cm [9]. Our patient had an ultrasound performed three days before delivery that continued to show persistent oligohydramnios for the ruptured Twin A. Adverse neonatal outcomes associated with PV-PPROM are related to chronic oligohydramnios. They may include pulmonary hypoplasia and limb deformities such as clubbed feet. The prevalence of pulmonary hypoplasia in neonates of pregnancies complicated by PV-PPROM is approximately 30%, and the mortality rate is approximately 70% to 90%. The incidence is inversely related to the gestational age at the time of membrane rupture [10]. About 14% of PV-PPROMs stop leaking amniotic fluid, which some say could be due to “resealing” of the fetal membranes. These pregnancies have outcomes similar to pregnancies uncomplicated by ROM [10]. In our case, the patient continued to report concerns of leaking of fluid throughout her antepartum stay with persistent oligohydramnios, and Twin A, the ruptured twin, was diagnosed with both pulmonary hypoplasia and positional congenital deformity of both feet upon delivery. Despite these morbidities, the record most recently indicates that Twin A was discharged home from the NICU after a 101-day stay in stable condition. As for maternal morbidity, postpartum endometritis occurs in approximately 40% of women with PV-PPROM, and, as noted, our patient developed this complication [10].

This report has several limitations. First, magnesium sulfate was used for tocolysis to administer a corticosteroid rescue course when the patient began to have contractions on day 113 after rupture. In a 2014 meta-analysis comparing magnesium sulfate against placebo, magnesium sulfate did not result in a statistical reduction in birth less than 48 hours after trial entry or improved neonatal and maternal outcomes [11]. Maximum efficacy of corticosteroids occurs two to seven days after administration of the first dose, and efficacy is incomplete less than 24 hours from administration with a decline after seven days. Our patient only received one dose of rescue course betamethasone, and this dose was given less than 24 hours before delivery. It may have been more advantageous for our patient to receive a different, more first-line approach for tocolysis to improve overall efficacy. Secondly, antibiotics may be considered as early as 20 0/7 weeks of gestation for PV-PPROM [12]. Our patient was admitted at 23 weeks for antenatal interventions (corticosteroids, antibiotics, magnesium sulfate for neuroprotection). It is unknown if latency antibiotics administered at 20 weeks would have made a difference in outcome as our patient never developed chorioamnionitis. Although antenatal interventions have improved outcomes for pregnancies greater than 24 weeks, most research has not studied their effectiveness in pregnancies less than 24 weeks, and, therefore, their effectiveness is unknown. Therefore, expectantly managing costs should be weighed against more aggressive management in these types of patients.

## Conclusions

We report an overall favorable outcome for a multifetal pregnancy complicated by PV-PPROM of one fetus at 13 weeks' gestation. Despite the overwhelming amount of data that suggest the prognosis for multifetal pregnancies with PV ROM is poor; it would seem our case is an exception to the rule. The authors believe this improved outcome was multifactorial such as young maternal age, no prior medical conditions or complications, and earlier gestational age of ROM which is associated with longer latency until delivery. In addition, the patient had good compliance, lived close to the hospital, and came in immediately if there were any concerns. To the best of our knowledge, this is the longest case of successful expectant management of both twins with PV-PPROM. Although just one case is insufficient to make any significant conclusions or provide recommendations, it highlights the need for further research regarding PV ROM, especially in multifetal pregnancies.
